# High Filling Pressures in HFpEF: Do Men and Women Get There Differently?∗

**DOI:** 10.1016/j.jacadv.2023.100399

**Published:** 2023-06-30

**Authors:** Yogesh N.V. Reddy

**Affiliations:** The Division of Cardiovascular Diseases, Mayo Clinic, Rochester, Minnesota, USA

**Keywords:** exercise hemodynamics, HFpEF

**Figure:**
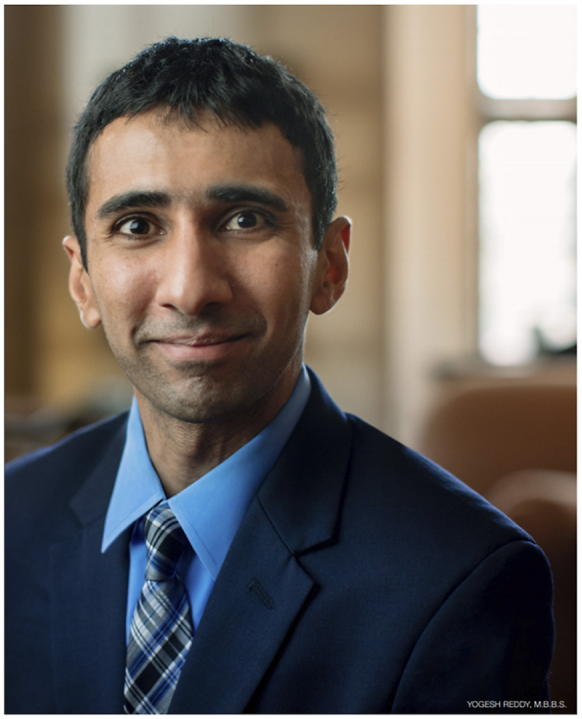

In the last 2 decades, we have learned that the pathophysiology of heart failure with preserved ejection fraction (HFpEF) is nothing short of complex. Abnormalities in global myocardial performance vascular reserve, and skeletal muscle all variably contribute to the exercise limitations in individual patients with HFpEF. In contrast to this heterogeneity in pathophysiology, there is homogeneity in disease manifestation where all patients with HFpEF universally have ‘pump failure’ reflected by an inability of the heart to deliver blood to the body during exercise without pathological increases in left atrial (LA) pressures. This abnormal rise in exertional LA pressure is the pathognomonic abnormality in HFpEF being associated with pulmonary edema,^1^ dyspnea,^2^ exercise capacity,^3^ and prognosis^4^; and responds favorably to SGLT2 inhibitors as a potential mediator of benefit as observed in the CAMEO-DAPA (Evaluation of the Cardiac and Metabolic Effects of Dapagliflozin in Heart Failure with preserved Ejection Fraction) trial.

In clinical practice, the diagnosis of HFpEF is established based on an increase in exercise pulmonary capillary wedge pressure (PCWP) [ie, LA pressure] to at least 25 mm Hg. The use of absolute PCWP elevation to diagnose HFpEF is based on the rationale that the lungs experience dynamic congestion related to the absolute hydrostatic pressure elevation in the pulmonary capillaries.^1^ Despite the fact that women on average have smaller hearts and height compared to men, we do not correct the abnormal PCWP cutoff for underlying heart or body size reflecting the fact that similar to arterial hypertension, LA hypertension is fundamentally abnormal regardless of how we get there. Despite the use of similar PCWP cutoffs to diagnose HFpEF in men and women, women with HFpEF have unique disease features including greater abdominal adiposity contributing to their hemodynamic abnormalities,^5^ and more limited myocardial and vascular reserve.^6^

In this issue of *JACC: Advances*, Schulz et al^7^ provide new insight into sex-specific impairments in cardiac reserve in HFpEF. This was a secondary analysis of 68 patients who underwent a sophisticated exercise cardiac magnetic resonance imaging and catheterization protocol with the diagnosis of HFpEF definitively established by an elevated PCWP at rest or exercise. There were 44 women (25 HFpEF, 19 controls) and 24 men (9 HFpEF, 15 controls). Compared to male HFpEF, female patients with HFpEF had smaller biventricular/biatrial volumes coupled with smaller left ventricular (LV) filling volume, biventricular stroke volume, and cardiac output during both rest and exercise. Notably, the smaller total heart volumes and cardiac output seemed to be an innate characteristic of female hearts being similarly present in female controls. Despite lower workload and less myocardial flow during stress in the smaller female HFpEF hearts (peak cardiac output [CO], 8.7 vs 13 L/min; *P* = 0.002), the absolute PCWP rose to similarly abnormal levels in both women and men (27 vs 26 mm Hg, *P* = 0.28), reflecting functionally worse left heart capacitance in female HFpEF. There was a sex-specific interaction with worse LA functional reserve only in female HFpEF, with an inability to effectively decrease LA minimal volume or improve LA filling during exercise. This dynamic LA filling impairment was likely related to a combination of an inherent LA myopathy and abnormal atrio-ventricular coupling—related in part to worse LV diastolic filling, worse vascular stiffness, and impaired LV strain during exercise which impacts atrial filling through annular descent. These LA reserve limitations were not seen in female controls or male HFpEF suggesting that loss of LA functional reserve may be a preferential driver of elevation in exercise PCWP in female HFpEF.

This elegant study provides unparalleled insight into 4 chamber myocardial flow and function with the unique combination of gold standard pressure and volume measures during exercise. As is evident from emerging literature,^8^^,^^9^ LA function appears to be a critical mediator of the elevation in PCWP during exertion in HFpEF, and the current study suggests it may be even more important to female HFpEF. Although the methods are highly sophisticated, the small sample sizes of men with HFpEF and controls may have resulted in insufficient power to detect LA functional reserve changes in men requiring further study. If validated, this may impact the reported utility of LA strain to noninvasively diagnose HFpEF in men with potential need for sex-specific cutoffs.^8^ The exclusion of patients with persistent atrial fibrillation who have the worst LA dysfunction likely underestimates the burden of LA myopathy to both men and women with HFpEF,^9^ and the study results are therefore only applicable to the subset of HFpEF in sinus rhythm.

This important study provides novel insight into the way male and female HFpEF hearts respond to exercise and achieve the abnormal PCWP elevation pathognomonic of the disease. The overall smaller LV/LA volumes and lower flow for similarly abnormal PCWP elevation suggest functionally worse capacitance of the left heart contributing to the abnormal exertional PCWP elevation seen in female HFpEF. Although some have advocated for correction of PCWP related to CO to diagnose HFpEF, this study raises caution with this approach given the generally lower cardiac outputs achieved in smaller female HFpEF hearts. As recently reported, the use of PCWP indexed to CO to diagnose HFpEF only appears to misclassify patients, and women in particular were more likely to be penalized and overdiagnosed with HFpEF with the use of exercise PCWP corrected for CO as opposed to using absolute PCWP.^10^

The present data suggest that therapies targeting improvement in ventricular capacitance and atrial operating compliance may hold preferential promise to improve exercise tolerance in female HFpEF. In terms of HFpEF prevention, long term high intensity exercise training has been shown to improve LV capacitance^11^ and may be particularly beneficial to prevent HFpEF in female patients with smaller hearts that are inherently more prone to develop abnormal exercise PCWP elevation. Given the abnormalities in atrial reserve observed in female patients with HFpEF who are in sinus rhythm, atrial fibrillation is likely to be poorly tolerated^9^ and the risk-benefit of catheter ablation for patients with paroxysmal atrial fibrillation requires further study stratified by sex. The abnormal LA compliance reserve in female patients with HFpEF may also preferentially respond to mechanical atrial level shunting, and it is notable that female patients appeared to benefit more than men in the REDUCE LAP-HF II (Atrial shunt device for heart failure with preserved and mildly reduced ejection fraction) trial. The poor heart capacitance in female patients with HFpEF may also underlie the prominent benefits of weight loss in female obese HFpEF, with improvements in quality of life and exercise capacity. Weight loss results in improvement in pericardial restraint and hemodynamics in obese patients without heart failure,^12^ but its impact on LA function is less clear,^13^ and further study of weight loss in female patients with HFpEF with obesity is urgently needed. The study by Schulz et al^7^ remind us that differences in the way men and women’s hearts respond to exercise in HFpEF may well prove to have critical therapeutic impact for current and future therapies being tested in clinical trials.

## Funding support and author disclosures

Dr Reddy is supported by National Heart, Lung, And Blood Institute of the National Institutes of Health (NHLBI) Award Number K23HL164901-01A1; grants from Sleep Number, Bayer, and United pharmaceuticals; and the Earl Wood Career Development Award from Mayo Clinic.
